# *APOE*ε4 Allele Modulates Region‐Specific Transcriptomic Changes in Epigenetic Control Pathways: Implications for Late‐Onset Alzheimer’s Disease Risk

**DOI:** 10.1002/alz.092722

**Published:** 2025-01-03

**Authors:** Nicole Delatorre, Hannah Van Rossum, Anna Parker, Roberta Diaz Brinton

**Affiliations:** ^1^ University of Arizona, Tucson, AZ USA

## Abstract

**Background:**

Age, sex, and APOE genotype are well‐established risk factors for late‐onset Alzheimer’s Disease (LOAD). Previous findings demonstrate that neuroinflammatory profiles of the human midlife female brain closely resemble the human AD brain. Given *APOE*’s role in LOAD pathogenesis, here we investigate the contribution of this risk factor on targeted AD relevant transcriptional pathways.

**Methods:**

Human corpus callosum, hippocampus, and hypothalamus tissue samples from 31 healthy control brain donors (n = 16 females; age = 48.4yrs ±3.7) without neurodegenerative disease and 20 LOAD (n = 10 females; age = 70.2 yrs±3.6) brain donors were obtained from the NIH NeuroBioBank (University of Maryland, University of Miami, and Mt. Sinai). *APOE* genotype was determined by electrophoresis and classified by the presence of *APOE*ε4 alleles (carriers *n* = 26). Transcriptomic analyses were conducted via NanoString nCounter neuroinflammation panel, resulting in 23 pathways. The log2 fold change of gene expression was calculated utilizing the Rosalind platform. Wherein ε4 carriers (condition) were compared to ε4 non‐carriers (control). Primary analyses compared *APOE*ε4 carriers to noncarriers. Whole pathway significance was determined using Fisher’s exact test.

**Results:**

*APOE*ε4 carriers exhibited significantly downregulated astrocyte function in the corpus callosum (p = 0.001), upregulated adaptive immune response in the hippocampus (p = 0.321), and upregulated epigenetic regulation in the hypothalamus (p = 0.007). Sub‐analyses based on sex, disease status (healthy vs. LOAD), and *APOE*ε4 status revealed consistent within‐sex differences only for epigenetic regulation. Specifically, male (p = 0.015) and female (p = 0.001) midlife *APOE*ε4 carriers differed significantly from non‐carrier counterparts in the hypothalamus. Significance of epigenetic regulation was also observed in AD between male and female *APOE*ε4 carriers and their non‐carrier counterparts both in the hypothalamus (p = 0.023; 0.016) and hippocampus (p = 0.032;0.001). No between‐sex differences were identified in any sub‐analyses.

**Conclusion:**

These results provide evidence of region‐specific differences in adaptive immune and epigenetic pathways driven by and specific for the APOEε4 allele. These targeted transcriptional analyses in post‐mortem brain indicate that at late‐stage AD, *APOE*ε4 is the principle differentiating factor in the transcriptional profile of targeted genetic pathways. Further, the data indicate activation of epigenetic control pathways within both the hypothalamus and hippocampus. The outcomes of these analyses support future studies to characterize epigenetic regulation in late‐onset Alzheimer’s disease.

